# The Potential of Spent Coffee Grounds in Functional Food Development

**DOI:** 10.3390/nu15040994

**Published:** 2023-02-16

**Authors:** Elza Bevilacqua, Vinicius Cruzat, Indu Singh, Roselyn B. Rose’Meyer, Sunil K. Panchal, Lindsay Brown

**Affiliations:** 1 School of Pharmacy and Medical Sciences, Griffith University, Gold Coast, QLD 4222, Australia; 2 Faculty of Health, Southern Cross University, Gold Coast, QLD 4225, Australia; 3 School of Science, Western Sydney University, Richmond, NSW 2753, Australia

**Keywords:** spent coffee grounds, chlorogenic acid, melanoidins, trigonelline, caffeine

## Abstract

Coffee is a popular and widely consumed beverage worldwide, with epidemiological studies showing reduced risk of cardiovascular disease, cancers and non-alcoholic fatty liver disease. However, few studies have investigated the health effects of the post-brewing coffee product, spent coffee grounds (SCG), from either hot- or cold-brew coffee. SCG from hot-brew coffee improved metabolic parameters in rats with diet-induced metabolic syndrome and improved gut microbiome in these rats and in humans; further, SCG reduced energy consumption in humans. SCG contains similar bioactive compounds as the beverage including caffeine, chlorogenic acids, trigonelline, polyphenols and melanoidins, with established health benefits and safety for human consumption. Further, SCG utilisation could reduce the estimated 6–8 million tonnes of waste each year worldwide from production of coffee as a beverage. In this article, we explore SCG as a major by-product of coffee production and consumption, together with the potential economic impacts of health and non-health applications of SCG. The known bioactive compounds present in hot- and cold-brew coffee and SCG show potential effects in cardiovascular disease, cancer, liver disease and metabolic disorders. Based on these potential health benefits of SCG, it is expected that foods including SCG may moderate chronic human disease while reducing the environmental impact of waste otherwise dumped in landfill.

## 1. Coffee Waste Products—From Farm to Landfill

**Figure 1:**
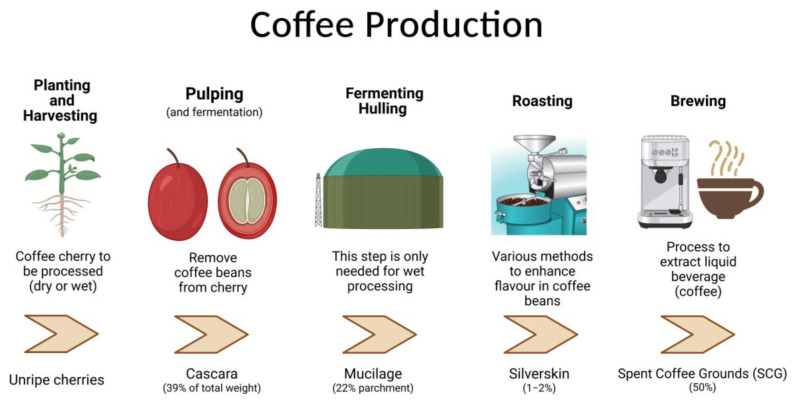
Coffee production generating waste. Data sourced from [14,17,18].

**Figure 2:**
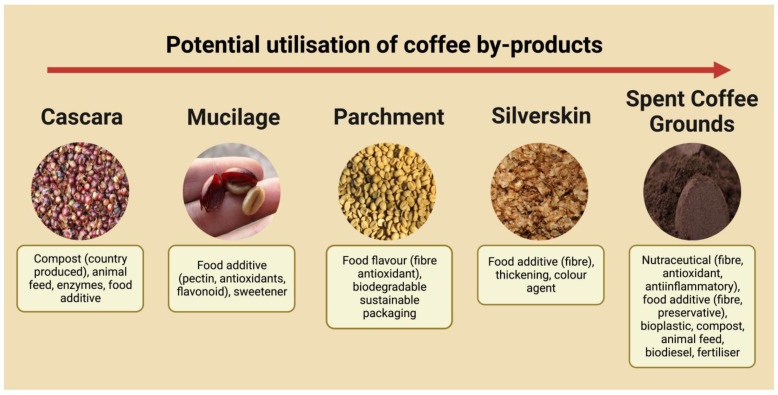
Coffee by-products and their potential applications. Data sourced from [14,17,18].

Spent coffee grounds (SCG) are the ultimate waste product in the consumption of coffee as a beverage. Coffee beverage consumption continues to have a remarkable economic, social and cultural impact across the globe [1,2]. Worldwide, the estimated coffee bean production in July 2020 to June 2021 was ~175 million 60-kg bags [3], approximately 10.5 million tonnes. Coffee production requires farming, harvesting, pulping of coffee cherry, fermentation and hulling in wet methods, roasting and brewing (Figure 1 and Figure 2) [4]. Significant wastage occurs during all stages of production, with the major impact being in developing countries, where most coffee is grown, such as Brazil, Vietnam, Colombia, Indonesia and Ethiopia. The economies of these countries rely heavily on coffee production. For example, in Brazil, more than 8 million people (around 4% of the population) are employed in coffee production by the coffee farms, excluding retail outlets, hospitality and other businesses involving commercialisation of coffee products [5]. Brazil’s coffee production constituted 5% of total export revenue at over USD 4.8 billion in 2019/2020 [6,7]. Therefore, the economic and environmental impacts of coffee farming are mainly observed in developing countries in tropical areas; these impacts include subsistence farming, heavy application of pesticides and fertilisers, destruction of complex tropical ecosystems, decreased soil fertility and contaminated water resources [8,9,10,11], which collectively can increase health risks for local communities [12,13]. The estimate of waste produced from coffee production worldwide is at least 6–8 million tonnes every year [14,15]. This value exclusively represents by-products of coffee production such as cascara, husk, mucilage, silverskin and post-brewing spent coffee grounds (SCG), and excludes contaminated soil and water [16].

The major coffee buyers are developed countries with large populations such as the USA, which imported about 29 million 60-kg bags (about 1.75 million tonnes) of coffee worth about USD 6.9 billion in 2021 [19]. The USA is one of many countries that produced very limited amounts of coffee, with production of 2270 tonnes in Hawaii and California in 2021 [20,21]. Post-consumption coffee waste includes SCG as well as increased slowly decaying microplastics [22] and nanoplastics [23] in the environment, with unknown long-term health risks. As an example, Planet Ark in 2016 in Sydney estimated that Australians use 1 billion coffee cups each year, producing 60,000 tonnes of plastic waste [24]. Therefore, unlike the waste products of coffee production, post-consumption pollution is mainly a problem of developed countries, the highest consumers. SCG production provides an increased 50–100% of the weight of the coffee beans as a standard double-shot espresso uses around 30 g of dry ground roasted coffee beans to yield 45–60 g of moist SCG [25]. The Planet Ark report in 2016 estimated that the 921 cafes in Sydney produced more than 3000 tonnes of SCG per year, with 93% ending up in landfill [24]. Thus, developing processes to use SCG in industrial or food production may be the more feasible approach to limiting the environmental and health damage from coffee waste, as these methods are more likely to be implemented in developed countries.

However, there are few studies investigating the health benefits of SCG, although there is a growing interest in its potential, due to the presence of many bioactive compounds that are also found in coffee beverages [26,27]. Our aim in this review is firstly to explore the production and identification of waste products in coffee production and consumption. Secondly, we will evaluate the potential health benefits of SCG, mainly using studies on coffee as a beverage and the compounds found in SCG. Thirdly, understanding the health benefits could allow the development of functional foods at affordable costs using SCG as an easily available product rather than complete purification of individual bioactive compounds. Functional foods provide health benefits beyond their nutritional values and thus have the potential to reduce the risk of chronic diseases. As an additional benefit, use of SCG in functional foods could reduce environmental damage by post-consumption coffee waste.

## 2. Bioactive Ingredients of SCG

The first step in value-adding to a complex mixture such as SCG is an understanding of the compounds present in the mixture, including the chemical changes during the process of roasting. By definition, the compounds present in SCG from roasted coffee powder are the compounds that have not been extracted during the production of the beverage. Low-molecular-weight bioactive compounds extracted into coffee as a beverage include caffeine, chlorogenic acids (CGA), trigonelline, tryptophan alkaloids and diterpenes such as cafestol and kahweol [28]. Further, coffee as a beverage produced by hot-brew processes contains approximately 1000 volatile organic compounds, which vary on growing and post-harvest conditions [29]. Cold-brew coffee differs from hot-brew coffee in the extent of compounds extracted during brewing rather than in the compounds that are present [30]. Roasting leads to the production of melanoidins by the non-enzymatic Maillard reaction which make up about 13–25% of the dry weight of the roasted coffee powder [31,32]. The chemical structures of the many melanoidins are only partly known, but the compounds include polysaccharides such as galactomannans and arabinogalactans, denatured proteins and CGA [31]. After roasting, coffee powder also contains carbohydrates (38–42%), proteins (8–14%), phenolic compounds (3–4%), lipids (11–17%), minerals (5%), fatty acids (3%), caffeine (1–2%) and trigonelline (1%) [29]. Degradation products during roasting include the carcinogen, acrylamide; practical solutions to reduce acrylamide production were proposed [33]. The SCG remaining after production of hot-brew coffee include carbohydrates such as 8–15% celluloses and 30–40% hemicelluloses, 20–30% lignins, 7–21% lipids and minerals, and 13–17% proteins, together with phenolic compounds (12 mg/g), caffeine (14.5 µg/g) and CGA (31.8 µg/g) [34]. Further, isolation and characterisation of these components were reviewed [34]. Similar information on SCG produced after cold-brew extraction processes is not available.

The chemical composition of coffee produced by either hot-brew or cold-brew processes will alter depending on factors including farming practices and extraction methods. The differences in concentrations between extraction processes implies that the content of bioactive compounds including caffeine and CGA remaining in SCG will depend on the extraction method used in the production of the beverage. The major compounds in coffee extracted by either hot-brew or cold-brew processes include caffeine, CGA, trigonelline and the diterpenes kahweol and cafestol [29,35,36,37]. Hot-brew processes with Ethiopian Arabica coffee showed highest caffeine and CGA concentrations in espresso coffees, up to 3–6 times higher than in Moka and filtered coffees [37]. The most efficient espresso methods used 14 g of fine powder and extraction for one minute at 93 °C and pressure of 9 bar [37]. The highest caffeine extraction from a 95% Robusta and 5% Arabica blend was in an espresso machine using 7.5 g of powder and 25 mL water at 92 °C and a pressure of 7 bar [35]. Unlike hot-brew coffee, cold-brew coffee extraction is a low-temperature, long-contact process, with different reported procedures to extract medium-roasted Arabica coffee under various conditions, such as using 50–100 g powder/L at 8 °C for 24 h [30] or 25 g powder/L for 282 min at 20 °C [35]. Extraction of caffeine and CGA by cold-brew procedures at room temperature reached a steady-state condition after around 400 min [36]. Further, per cup caffeine and CGA contents were greater in cold-brew processes than hot-brew espresso coffees [37]. The highest scores in sensory evaluation of cold-brew coffee, characterised by strong sweetness, fruity and floral flavours, medium bitterness and acidity, and a creamy body, were found after a 14 h extraction of coarse ground medium-roasted coffee at room temperature of 20 °C [38]. Cold-brew coffee showed increased floral flavour when compared to hot-brew coffee, and hot-brew coffee exhibited increased bitterness, sour taste and rubber flavour [39]. The differing results from different extraction procedures means that it is not possible to extrapolate the daily doses of caffeine and CGA from the number of cups of coffee consumed per day [37].

## 3. Value-Adding to SCG Outside the Health Industry

Utilisation of coffee by-products including SCG by industry has been a worldwide topic of research [14]. As a valuable industrial resource [27], industrial uses may provide the expertise in purifying compounds from SCG for therapeutic studies and also provide the financial support for these studies. This section will summarise some of the potential industrial uses of SCG including animal feed, biofuels, nutraceutical, cosmetic, fertilisers, composting and biopesticides [17,40,41].

Raw materials from coffee waste such as polysaccharide-rich fraction in SCG provide viscous and stable liquid solutions that are suitable for use as raw materials for biodegradable films or coating for packaging [42,43]. Such alternative materials for non-biodegradable fossil fuel-derived plastic packaging have been the target of research as many countries make the commitment to replace plastic packaging with environmentally friendly bioplastics by 2030 [44,45]. The biodegradable industry is growing, worth over USD 250 billion in 2020 and is expected to reach over USD 380 billion by 2028 [46].

SCG contain nitrogen and other important minerals required in both compost and fertilisers, and so could be used by the agriculture industry [4,14,27,47]. The current high costs of agricultural fertilisers, including nitrogen fertilisers, and compost prices at around 1320 AUD/tonne are expected to keep increasing [48]. Nutrient density increases in coffee plantations have been reported using SCG as part of fertilisers [49]. Potentially toxic responses to SCG to soil due to caffeine and high amounts of antioxidants [50] can be decreased by farming earthworms to decrease the caffeine content of SCG [51,52], making SCG then usable for the composting and fertilising industry.

Biofuels such as bioethanol, biogas and biodiesel can be produced from SCG, thus redirecting large amounts of coffee waste as a sustainable source of biofuel [53]. The commitment to reduce and eliminate fossil fuel use by generating renewable energy resources is the focus of almost the entire world [54]. The oil (10–30% of dry weight of SCG) extracted using ultrasound from SCG has the potential to be re-utilised to produce biodiesel [18,55,56]. Further, hydrothermal liquefaction of SCG has also been investigated as a viable option for producing crude bio-oil without the need of oil extraction [57]. In addition, fermenting the remaining oil-free SCG carbohydrate compounds showed the potential to use this waste to produce bioethanol [55]. Research findings are very promising, even though the methods to produce biofuels from SCG need improvements in scalability and efficiency [14].

SCG may be an efficient, low-cost and certainly environmentally friendly source of antioxidants, polyphenols and biomaterials for pharmaceutical products [26,58]. These compounds have been tested in cosmetics as anti-ageing and protective agents such as sunscreens, natural fillers and preservatives [58,59].

SCG, along with other coffee plant wastes, have been successfully used as a sustainable, cost-effective and healthy food additive in baked products, granolas, slow-cooked meals, seasoning for barbecues and desserts [60,61,62,63,64]. The production of food additives is a growing industry that turns over close to USD 45 billion a year [65]. SCG have been used in the preparation of baked products such as cookies and cakes as well as the production of beverages including alcoholic beverages [66]. Cookies prepared using SCG showed presence of caffeine, phenolic acids and polyphenols such as CGA [63]. Further, CGA extracts from coffee have been used in fried doughnuts, soymilk, wheat bread, liquid Khask, dark chocolate, yogurt and even instant coffee, which may increase the health benefits of these foods [67]. Increasing attention towards using coffee waste as a food additive will help to provide sustainable economic options to coffee farmers and reduce environmental impacts [66,68].

As SCG are a good source of caffeine, polyphenols such as CGA and melanoidins, it can be used as a raw material for the isolation of these compounds. The recovery of these compounds from SCG has used a range of methods of extraction [69,70]. Some of these methods include conventional solvent extraction, high hydrostatic pressure-assisted extraction, ultrasound-assisted extraction and microwave-assisted extraction [69,70,71,72]. Further, extraction methods have also been studied for coffee oil, which is a rich source of fatty acids and caffeine [73,74]. SCG after coffee oil extraction can be used for the extraction of galactomannan, diterpenes and mannose [74]. The leftover material can then be fermented into bioethanol [74]. This scheme, termed as biorefinery, can generate many valuable components from the SCG biomass that is generally being discarded in landfill, potentially generating many avenues for generating commercially viable components [41]. Thus, biorefinery using SCG can provide many valuable products including biodiesel, hydrocarbon fuel, bio-hydrogen, glycerine, many pharmaceutical-grade bioactive compounds, bioethanol, bio-oil, biochar, polymers and biogas [75].

## 4. Health Benefits of SCG

**Figure 3:**
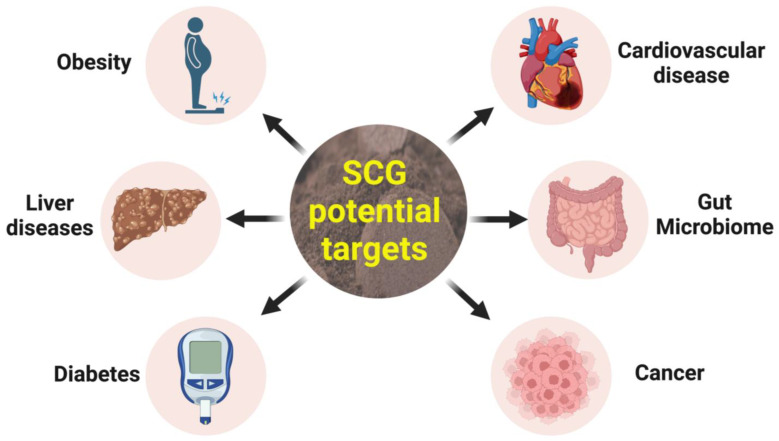
Potential health benefits of spent coffee grounds.

Although there are relatively few studies describing the physiological effects of SCG, they suggest that SCG intake may improve health and is safe. We have reported that modulation of gut microbiota by SCG from a hot-brew process, probably by melanoidins, reduced body weight, abdominal fat mass, systolic blood pressure and plasma triglycerides, improved glucose tolerance and improved the structure of the heart and liver in a rat model of diet-induced metabolic syndrome [76]. Coffee and SCG have been linked with changes in gut microbiota including increases in *Bifidobacterium* and decreases in *Clostridium* and *Escherichia coli* [77,78,79,80,81]. Further, CGA from coffee has shown non-polysaccharide-based prebiotic effects in an in vitro study through selective growth of human faecal microbiota [82]. These beneficial changes can help in improving the short-chain fatty acid profile produced by the gut microbiota and hence improve their composition and function. Pilot human studies found that consuming cookies enriched with SCG containing prebiotics promoted short-term satiety and reduced overall energy consumption even without other lifestyle changes [83]. Studies with SCG in humans include a small, randomised control single-blind parallel-group study and a pilot crossover randomised single-blind control study. Both studies observed better outcomes when participants ingested an extract of SCG antioxidant fibre. However, SCG (as a whole) also showed positive effects when compared to placebo [83,84]. An in vitro study suggested that SCG prebiotic fibre increased short-chain fatty acid production, resulting in gut microbiota modulation [85]. A small human clinical trial looking at chronotype and circadian locomotor activity in young adults found that the consumption of antioxidant fibre from SCG improved quality and length of sleep associated with an increased fermentation in the colon and short-chain fatty acids [84]. Furthermore, the inclusion of SCG with gluten-free flour (rice) in cookies improved sensory acceptance, with higher nutritional value as a source of fibre and polyphenols [86]. Figure 3 summarises the potential health benefits based on existing experimental evidence on SCG.

## 5. Health Benefits of Compounds in SCG

Bioactive compounds found in SCG have been researched for over 20 years, presenting evidence on the therapeutic effects when sourced from coffee [87,88]. Health benefits associated with the consumption of these compounds are directly associated with dose and frequency as well as source of compounds (for example, isolated pure compound vs. compound in coffee form). A summary of findings and types of studies is presented in Table 1. This section clarifies existing evidence on the compounds present in SCG concluding the health benefits associated with the consumption of SCG.

### 5.1. Chlorogenic Acids (CGA)

CGA is an abundant polyphenol found in plant foods. Coffee is the major source of CGA for humans, with amounts varying from 0.5–6 g to 100 g of dry coffee prior to the brewing process [36]. CGA is also present in SCG and preliminary experiments using SCG indicates compound activity [87,88].

When sourced from coffee, CGA affects cardiovascular health, glucose metabolism and obesity [36,89,92,93,96,98,112,113,114]. Similar findings from research using SCG are presented in Section 4. Overall, the main actions associated with CGA are antioxidant and anti-inflammatory.

Effects on cardiovascular health include potential benefits in regulating blood pressure, endothelial function and dyslipidaemia [36,89,92,93,112]. Specific mechanisms of action by which CGA may have a direct effect on blood pressure and endothelial function include increases in nitric oxide (NO) bioavailability by inhibiting reactive oxygen species (ROS), NADPH oxidase and superoxide anion generation [93]. Other mechanisms of action to improve cardiovascular risk factors such as dyslipidaemia include increased uptake of fatty acids in the liver and reduction in plasma low-density lipoprotein cholesterol in both animal and pilot human studies [93,115].

CGA effects on glucose metabolism may provide an alternative and non-invasive approach for the treatment and prevention of chronic metabolic diseases such as type 2 diabetes [112]. CGA reduced fasting blood glucose concentration in patients with impaired glucose tolerance at various doses and treatment duration [94,95]. CGA may act similarly to metformin, one of the most commonly prescribed pharmaceutical drugs for type 2 diabetes, as an insulin sensitiser [116]. Mechanisms of action for CGA to assist glucose metabolism include improving intestinal and adipocyte glucose absorption, potentially decreasing plasma glucose concentrations as well as influencing the glycaemic impact of foods and release of carbohydrate-specific digestive enzymes [95,117].

CGA decreased obesity, inhibited in vivo lipase enzymatic action and prevented lipid absorption [96,98,113,114]. In rats, CGA improved body weight, visceral fat accumulation and liver function, and decreased inflammatory cell infiltration in obese, hypertensive rats fed a high-fat and -carbohydrate diet [97]. In humans, a reduction in body weight and most markers associated with obesity, glucose and lipid metabolism were reported [114]. Further studies are needed to compare the difference in activity and concentration of CGA from different sources, including coffee (beverage), SCG, and their different methods of extractions (hot or cold brew), since temperature maybe relevant to this compound.

### 5.2. Caffeine

Caffeine is widely known for its mild stimulant effects, temporary energy boosts and sometimes changes in mood [118]. Caffeine absorption occurs 30–45 min after consumption and blood concentrations may take up to two hours to rise [119]. Caffeine is predominantly ingested as coffee in our diet, with an average double-shot coffee providing around 150 mg [118,120], and also found in SCG [88].

Similar to CGA, caffeine can impact cardiovascular health. The effect of caffeine in cardiovascular health depends on factors such as dose, time ingested, absorption variation and hepatic metabolism [121]. The mechanisms of action in which caffeine affects the cardiovascular system can include a reduction in cytoplasmic calcium concentrations in the vascular smooth muscle cells through cyclic adenosine monophosphate and an increase in the same in the endothelial cells favouring the endogenous synthesis of NO [121]. The main cardiovascular effect of caffeine is the increased concentration of NO, and, consequently, vasodilation.

The effects of caffeine in the nervous system are widely researched. One of the mechanisms of action in which caffeine affects the brain is by antagonising adenosine receptors, increasing the release of excitatory neurotransmitters such as glutamate and noradrenaline [122,123]. Caffeine potentially improves cognitive symptoms and has protective characteristics in neurodegenerative diseases such as Parkinson’s disease [99,123,124,125,126,127].

A widespread concern with caffeine is potential negative effects in those with existing cardiovascular conditions [128,129]. However, consumption of up to six cups of caffeinated coffee a day was not associated with an increased risk of cardiovascular outcomes, even with those who have history of hypertension and other cardiovascular diseases [120]. In addition, a meta-analysis showed those who consume three to five cups of caffeinated coffee a day have lower incidence of coronary artery disease, stroke and death due to cardiovascular causes [130]. However, longer-term or overconsumption of caffeine may cause addiction, insomnia, migraine and other adverse effects [131].

### 5.3. Trigonelline

Trigonelline is a pyridine alkaloid compound and a methylation product of vitamin B_3_, niacin [132], found in plant foods such as barley, cantaloupes, corn, onions, soybeans, tomatoes, peas, fenugreek seeds, coffee and SCG [133]. A 250 mL volume of brewed coffee provides 27 mg of trigonelline [134]. Higher concentrations of trigonelline are found in green coffee beans from the *C. arabica* species, and trigonelline changes into *N*-methylpyridinium and nicotinic acid during roasting [135,136].

In the nervous system, trigonelline improves the function of specific neurons, and sometimes the potential to regenerate certain neurons. Therefore, it is a possible intervention for neurovegetative diseases which are now incurable [102,103,107,137]. β-Amyloid peptide accumulation is a common risk factor and cause of Alzheimer’s disease [138]. The similarity of trigonelline to cotinine, an anti-Alzheimer’s drug, pushed studies that checked whether trigonelline had any affinity to interact with β-amyloid peptide, and results were promising [139]. Trigonelline was effective in suppressing oxidative stress, astrocyte activity and inflammation to prevent neuronal loss in the hippocampus to alleviate Alzheimer’s disease in mice [102]. Neuroinflammation is also a contributor to Alzheimer’s disease [140]. An animal study showed the anti-inflammatory effects and improvement of memory of trigonelline against liposaccharide-treated adult mice brains [104]. The positive results could be due to higher concentrations of brain-derived neurotrophic factor, lowered oxidative stress and decreased concentrations of tumour necrosis factor α, interleukin 6 and acetylcholinesterase [104]. Recently, a comprehensive animal study confirmed that trigonelline recovered memory function in a mouse model of Alzheimer’s disease [103]. The anti-Alzheimer’s disease effects of trigonelline in this study were confirmed by the reconstruction of neuronic networks after brain damage.

### 5.4. Cafestol and Kahweol

Cafestol and kahweol are the main diterpenes and their content is about 15% of total lipids in coffee [141]. Kahweol is largely found in *C. arabica* beans, whereas 16-O-methylcafestol ester is found mainly in *C. robusta* [142]. However, cafestol is found in both *C. arabica* and *C. robusta* [143]. Coffee consumption was associated with elevated serum cholesterol concentrations due to the presence of cafestol and kahweol esters [144,145]. Diterpenes are extracted from coffee during the brewing process, and when coffee is filtered, diterpenes are almost completely removed. In SCG, the presence of these compounds will also depend on the preparation method.

There are few data on the bioavailability and pharmacokinetics of cafestol and kahweol, especially in diseases, with most data from healthy individuals. An estimated 30% of cafestol is broken down in the stomach by gastric juices, with the remaining 70% absorbed in the duodenum at a rate of 84–93% [146]. Kahweol has a similar absorption rate and was absorbed in the small intestine at a higher rate of 91–95% [146].

Most of the evidence on these compounds’ health benefits is related to their ability to suppress activity, migration and proliferation of cancer cells. Kahweol acetate and cafestol inhibited proliferation and migration of prostate cancer cells, where other coffee compounds did not show the same effect [147]. The synergistic effects of both compounds may allow lower concentrations of these compounds to be effective in inhibiting prostate cancer progression. These findings could be important for those consuming unfiltered coffee, as concentrations of diterpenes are much higher than in filtered coffee. The mechanisms of actions were described as an ability to induce apoptosis and suppression of the epithelial–mesenchymal transition, and a reduction in androgen receptors and chemokine receptors CCR2 and CCR5, preventing cancer cell migration and proliferation [147]. 

Anti-angiogenesis activity from cafestol and kahweol has been published with experiments in vitro and protective effects in cancer proliferation and migration in endothelial cells. Angiogenesis plays an important role in cancer cell proliferation and migration [148].

Other health benefits of cafestol and kahweol include anti-diabetic and anti-inflammatory activity [149]. These compounds show anti-diabetic actions by increasing insulin secretion and glucose uptake by skeletal muscle, and AMP-activated protein kinase activation, which mimics metformin action [150,151]. Both compounds showed the ability to inhibit inflammatory mediators such as prostaglandin E_2_ and NO synthesis in lipopolysaccharide-activated macrophages, thus indicating their anti-inflammatory activity [152].

### 5.5. Melanoidins

Melanoidins are nitrogen-containing polymers produced during the non-enzymatic browning Maillard reaction, making these compounds a differentiation marker between green and roasted coffee beans present in coffee beverages and SCG. Melanoidins are not unique to coffee or SCG as other foods such as bread, roasted cocoa and beer undergo a Maillard reaction during preparation to produce melanoidins [153]. Hot-brewed coffee is likely to be the main source of melanoidins in the human diet [154]. Melanoidin concentrations in coffee may vary in roasted coffee beans, making around 25% of dry weight or slightly higher in a darker roasting process and around 29% in brewed coffee [155]. Melanoidins provide specific characteristics to foods such as flavour and brown colour [31]. Published biological activities of melanoidins include antioxidant, antimicrobial, ability to change xenobiotic enzymatic activity, prebiotic fibre and antihypertensive actions [153].

A recently published study concluded that melanoidins from coffee also undergo minor digestion in the upper gastrointestinal tract [156]. Melanoidins can be fermented by gut bacteria and produce short-chain fatty acids, modulating the bacterial population. This fermentation may also release phenolic compounds which can then be absorbed, increasing phenolic absorption from foods containing melanoidins. Modulation of gut bacteria by short-chain fatty acid production reduced symptoms of metabolic diseases [157,158].

The potential antioxidant activity of melanoidins on human health has been linked with protection against oxidative damages and it has been highly related to degrees of roasting [159]. Their ability to bind undesirable dietary metals also prevents oxidative damage [160]. High-molecular-weight fractions of coffee were able to completely inhibit lipid peroxidation in rat liver microsomes [161]. However, once isolated compounds were tested, they failed to duplicate the protective action alone, so two non-melanoidin compounds may be responsible to protective actions. In different in vitro methods, Maillard reaction products such as melanoidins may have similar antioxidant compounds to pre- or light-roasting polyphenol compounds found in coffee against oxidation from human low-density lipoproteins [155]. There are limited data published on in vivo antioxidant effects of coffee consumption, which is not specifically linked to melanoidins’ individual effect as it would also include polyphenols in coffee such as CGA. The antioxidant effects of roasted and brewed coffee were mainly attributed to melanoidins, as other antioxidant compounds in coffee are decreased by heat from roasting and brewing processes [162]. Other applications for melanoidins originating from foods other than coffee such as antioxidant and modulator of Phase I and II enzymes for detoxification were briefly described in a review, which could be applicable to coffee [162].

Although there is robust evidence on these compounds when sourced from coffee, studies to analyse each compound and their biological activity when sourced as part of SCG are needed. Understanding the biological responses of the compounds including caffeine, CGA, trigonelline, polyphenols, melanoidins and other antioxidants when sourced from SCG, rather than coffee (beverage), may provide options to test for therapeutic benefits as these compounds. SCG may be a sustainable resource for bioactive compounds with established health benefits and safety and efficacy for human consumption [18,27,88]. However, SCG are currently not utilised to the full potential.

## 6. Conclusions, Challenges and Future Directions

SCG can contribute to a wide variety of sustainable products, including animal feed, biofuels, fertilisers, compost and biopesticides. However, SCG applications can go beyond non-health-related purposes due to the presence of bioactive compounds in potentially therapeutic doses, such as CGA, caffeine, trigonelline, cafestol, kahweol and melanoidins. SCG, similar to hot-brew coffee, containing these compounds has potential to attenuate well-known metabolic disorders, including NAFLD, type 2 diabetes and cardiovascular disease.

This review shows that the literature on responses to SCG as a functional food component is often preliminary, so many more studies are required to understand what other compounds are present in coffee by-products, especially SCG, and how these could benefit human health. Moreover, it is important to elucidate the precise mechanisms by which the bioactive compounds obtained from SCG support health-related effects. As with other functional foods, SCG may experience challenges in clinical studies [163]. Some of these challenges may include limited industry funding to support the study, unsuitable placebos, maintaining food products and their safety, and limited opportunities to check compliance [163]. Thus, more focus on food by-products such as SCG from industries and funding bodies will help in making initial steps towards obtaining suitable clinical data that can be used for appropriate translation of the clinical outcomes.

As studies on SCG are limited regarding their health benefits, it is important to understand the differences between hot or cold extraction methods as well as differences in responses of these compounds when consumed in coffee beverages and SCG. Ultimately, more scientific investigations can promote economic and health benefits worldwide. The change from considering SCG as a waste product to one with widespread health and industrial uses could benefit both coffee producers and consumers. Finally, SCG as a low-cost raw material can provided affordable functional food products when used directly. However, purified nutraceutical product development can dramatically increase the cost of the process and hence the product, thus suggesting SCG as the most viable and affordable functional food option.

Limitations of current studies include the precise characterisation of the components of cold-brew coffee and SCG as well as longer and larger clinical trials in patients with chronic diseases. Further, limited attempts have been made to extract bioactive compounds from SCG for developing nutraceuticals. These nutraceuticals can serve as a viable option for the longer-term option for human consumption as supplements. Large-scale epidemiological or clinical studies in people consuming these supplements from SCG will be important in confirming any reduction in disease risk.

## Figures and Tables

**Table 1:** Studies analysing the components of spent coffee grounds and their benefits.

Compound	Type of Study	Findings
Chlorogenic acid	Human	Regulated blood pressure [89,90]; improved insulin secretion, uptake of glucose by intestinal cells [91]; improved insulin secretion [91]; improved dyslipidaemia and endothelial function [92,93]; improved fasting glucose in patients with impaired glucose tolerance [94,95]; body weight reduction and waist circumference reduction [95]
	In vitro	Improved lipase reaction [96]
	Animal	Reduced accumulation of fat in the liver and reduced blood lipids [97,98]; improved body weight and reduced visceral fat [97]
Caffeine	Human	Improved cognitive health in patients with degenerative disease [99]; better performance in tests in age-related cognitive impairment [100]; enhanced memory and cognitive performance in young adults [101]
Trigonelline	Animal	Improved specific neuron function [102]; improved memory in Alzheimer-induced mice [103]; suppressed oxidative stress and inflammation in the brain [102,104]; reduced blood glucose and improved lipid in metabolically ill animals [105,106]
	In vitro	Promoted regeneration of neuronal network by neurite outgrowth [107]
Melanoidins	In vitro	Antioxidant activity [108]; antibacterial activity against Gram-negative and Gram-positive bacteria [109]; antioxidant activity and activation of other gene-protective mechanisms in different cell lines [110]
	Ex vivo	Antioxidant activity and activation of other gene-protective mechanisms in human gut tissue [110]; fermentation of gut bacteria, activation of antioxidant pathways and modulation of gut bacteria population [111]

## Data Availability

Not applicable.
